# Clinical Application of Different Vertical Position Tests for Posterior Canal-Benign Paroxysmal Positional Vertigo-Cupulolithiasis

**DOI:** 10.3389/fneur.2022.930542

**Published:** 2022-07-12

**Authors:** Wenting Wang, Shuangmei Yan, Sai Zhang, Rui Han, Dong Li, Yihan Liu, Ting Zhang, Shaona Liu, Yuexia Wu, Ya Li, Xu Yang, Ping Gu

**Affiliations:** ^1^Department of Neurology, The First Hospital of Hebei Medical University, Shijiazhuang, China; ^2^Department of Vertigo Center, The First Hospital of Hebei Medical University, Shijiazhuang, China; ^3^Department of Neurology, Aerospace Center Hospital, Peking University Aerospace School of Clinical Medicine, Beijing, China; ^4^Brain Aging and Cognitive Neuroscience Laboratory of Hebei Province, Shijiazhuang, China

**Keywords:** posterior semicircular canal, nystagmus, cupulolithiasis, vertigo, Dix-Hallpike test, benign paroxysmal positional vertigo

## Abstract

**Background:**

Posterior canal-benign paroxysmal positional vertigo-cupulolithiasis (PC-BPPV-cu) is a new and controversial type of benign paroxysmal positional vertigo (BPPV). At present, there are few relevant clinical studies as to whether the Half Dix-Hallpike test (Half D-HT) induces more obvious nystagmus than the Dix Hallpike test (D-HT) and straight head hanging test (SHH) in patients with PC-BPPV-cu.

**Objectives:**

To investigate the clinical characteristics of PC-BPPV-cu, and analyze the diagnostic significance of the Dix-Hallpike test (D-HT), Half D-HT, and straight head hanging (SHH) test in these patients.

**Methods:**

A total of 46 patients with PC-BPPV-cu were enrolled, and divided into two groups (*N* = 23): a group A (induction order: D-HT, Half D-HT, SHH) and a group B (induction order: Half D-HT, D-HT, SHH).

**Results:**

Among 46 patients with PC-BPPV-cu, the bilateral and unilateral abnormality rates of the disease side were 5 cases and 41 cases, respectively. There were significant differences in the proportion of torsional-upbeating nystagmus and upbeating nystagmus among the three headhanging positions in 46 patients with PC-BPPV-cu (*P* < 0.001). The slow phase velocity (SPV) of induced nystagmus at half D-HT supine position was slower than D-HT supine position (*P* < 0.05) and SHH supine position (*P* < 0.05). The nystagmus latency of D-HT supine position was significantly shorter than half D-HT (*P* < 0.05) and SHH (*P* < 0.05). PC-BPPV-cu patients were accompanied by 53.5% semicircular canal paresis, 69.6% audiological abnormalities, 76% cervical vestibular evoked myogenic potential (cVEMP), and 75% video head impulse test (vHIT) abnormalities, the concordance rates of the four detection methods were similar (χ^2^ = 0.243, *P* = 0.970).

**Conclusions:**

The Half D-HT is simple and feasible, but might have a risk of false-negative diagnoses of the torsional-upbeating nystagmus and upbeating nystagmus. The D-HT is still a classic induction method for PC-BPPV-cu. The two complement each other and may aid in the diagnosis of PC-BPPV-cu patients. Future clinical applications of Half D-HT require extensive research to determine its diagnostic efficacy.

## Introduction

Benign paroxysmal positional vertigo (BPPV) is one of the most common episodic vestibular diseases, arising from a problem in the inner ear (1). Symptoms are repeated and brief periods of vertigo with movement, characterized by a spinning sensation upon changes in the position of the head (2). When the head moves to a certain position, it can induce transient vertigo, accompanied by nystagmus and autonomic nerve symptoms (3). Approximately, 2.4% of people are affected at some point in time (1). BPPV is always found in people between the ages of 50 and 70, affecting women twice as often as men (4). The most frequently involved semicircular canal was the posterior semicircular canal (80–90%), followed by the lateral semicircular canal (10%), while the least involved semicircular canal was the superior semicircular canal (2%). The lifetime prevalence rate was approximate 3–10%.

In 2015, the international Barany society put forward the latest diagnostic criteria for BPPV (5). Posterior canal benign paroxysmal positional vertigo (PC-BPPV) is the most common type of BPPV (6). According to the pathophysiological mechanisms, PC-BPPV is divided into posterior canal benign paroxysmal positional vertigo canalithiasis (PC-BPPV-ca) and posterior canal-benign paroxysmal positional vertigo-cupulolithiasis (PC-BPPV-cu). Its nystagmus is characterized by upbeating and torsional nystagmus in the position-induced test, and the duration is less or more than one min. The golden diagnostic standard of PC-BPPV-ca is the Dix-Hallpike test (D-HT). PC-BPPV was diagnosed if vertical upbeat nystagmus with or without torsional component was induced by the Dix-Hallpike test, and the reversal of the nystagmus often occurred when returning to an upright position; if vertical upbeat nystagmus with torsional component was induced, the torsional component involved the beating of the upper pole of the eyes toward the affected side (7). PC-BPPV-cu is one of the new and controversial BPPV in the BPPV diagnostic criteria proposed by the International Barany Society. It refers to the appearance of upbeating and torsional nystagmus lasing ≥1 min in the position-induced test. Epley (8) proposed that the Half Dix-Hallpike test (Half D-HT) can cause the affected crest cap of PC-BPPV-cu patients to produce a greater degree of deflection under the action of gravity, thereby inducing strong positional nystagmus with a short or no incubation period.

As PC-BPPV-cu is one of the new and controversial BPPVs in the BPPV diagnostic criteria, the clinical characteristics of PC-BPPV-cu are not well-investigated. Additionally, the diagnostic efficiency of Half D-HT in comparison with D-HT and straight head hanging test (SHH) is rarely reported. This study intends to explore the clinical characteristics of PC-BPPV-cu and evaluate its nystagmus characteristics by different vertical position tests, so as to help the treatment of PC-BPPV-cu.

## Patients and Methods

### Patients

A total of 46 patients with PC-BPPV-cu were collected in the Neurology Department and Vertigo Center of the First Hospital of Hebei Medical University from February 2019 to February 2022. The study was approved by the Ethics Committee of the First Hospital of Hebei Medical University. Written informed consent was obtained from each patient.

Among the patients, there were 20 men and 26women. Detailed basic information and medical history including gender, age, and current medical history (including duration, onset of seizure, duration of onset, triggers, motivating factors, accompanying symptoms), smoking, drinking history, and past history (including high blood pressure, diabetes, hyperlipidemia, coronary heart disease, cerebral infarction, migraine, brain injury, etc., Meniere's disease, vestibular neuritis, otitis media, osteoporosis, mood, and sleep disorders was) recorded. All patients underwent routine neuro-otology examination. Dynamic position tests included D-HT, Half D-HT, and SHH. Auxiliary examinations include pure tone audiometry, eye movement tests, canal paresis (CP) of caloric test, vestibular evoked myogenic potential (VEMP) (Interacoustics, Middelfart, Denmark. Model: Eclipse), video head impulse test (vHIT) (Interacoustics, Middelfart, Denmark. Model: EyeSeeCam) and video-nystagnography (VNG) (Interacoustics, Middelfart, Denmark. Model: V0425). If necessary, head computed tomography (CT), magnetic resonance imaging (MRI), CT angiography (CTA), CT perfusion (CTP) examinations were performed.

### Methods

The diagnosis of PC-BPPV-cu refers to the latest BPPV diagnostic criteria proposed by the International Barany Society in 2015 (5), and the diagnosis of PC-BPPV-cu is based on the patient's typical nystagmus and its clinical manifestations: (1) D-HT and/or Half D-HT induces vertical nystagmus with torsion component. Nystagmus was often reversed when sitting up. If the vertical nystagmus was accompanied by a torsion component, the twisting direction of the upper pole of the eyeball was toward the affected side. (2) The duration of nystagmus was ≥1 min. The random number table method was used to divide 46 patients into two groups (*N* = 23): group A (induction order: D-HT, Half D-HT, SHH) and group B (induction order: Half D-HT, D-HT, SHH). A G-Force BPPV diagnosis and treatment instrument (Product model: GYZ-ZLY-I. China Medical (TianJin) Group Co., LTD., China) was used to simulate the position tests were conducted. A 5-min rest between each position induction test was set to eliminate the fatigue. Three vertical position induction test methods were set as follows: (1) D-HT: The patient sits upright on the G-Force BPPV diagnosis and treatment instrument, head turned 45° to one side, and at the same time the patient is made tolie down and the head hang back at 20° below the horizontal plane. Vertigo and nystagmus were recorded until the nystagmus disappeared. The patients were quickly returned to the sitting position, followed by the check of the opposite side in the same way. (2) Half D-HT: The patient sits upright on the G-Force BPPV diagnosis and treatment instrument, head turned to one side by 45°, and then lies back to the supine position (~30° tilted from the horizontal front). The patient's vertigo and nystagmus from this position were recorded, until the nystagmus disappeared and quickly returned to the sitting position, and then checked the opposite side in the same way. (3) SHH: The patient sits on the G-Force BPPV diagnosis and treatment instrument, lies down quickly, and hangs their head vertically at least 30° below the horizontal plane. The patient's vertigo and nystagmus at this position were recorded until the nystagmus disappeared. After disappearing, the patients were quickly returned to the sitting position. The G-Force BPPV diagnosis and treatment instrument was used to determine and record nystagmus. The subject was fixed on a swivel chair, wearing a high-definition 1080P eye video mask, in which the chair can complete more than 360° rotating motion and the display screen can clearly show the direction of eye movement and nystagmus parameters. In the position test, the maximum slow phase angular velocity of the nystagmus in the horizontal or vertical direction of the induced nystagmus in the postural position is used as the slow phase velocity (SPV) value. Positional nystagmus diagnosis of PC-BPPV-cu was observed through a video-oculography (Product model: GYZ-ZLY-I. China Medical (TianJin) Group Co., LTD., China) according to the manufacturer's instructions.

### Statistical Analyses

SAS 9.3 software was used to analyze the data. The test results of a caloric test, audiology, cVEMP, vHIT, and the disease side were described in a cross table. The Chi-Squared test was used for comparison of count data between groups, and the Yates continuity correction or Fisher's exact test was performed if necessary. When the measurement data confirmed the normal distribution, the Student's *t*-test test was used for comparison between the two groups, and the Wilcoxon two-sample test was used when the measurement data did not conform to the normal distribution. For the comparison of measurement data among the three groups, one-way ANOVA analysis was used when the data were in a normal distribution. Otherwise, the Kruskal-Wallis test was used, and Student–Newman–Keuls (SNK) test was used for the comparison afterward. A *P* < 0.05 was considered to be statistically significant.

## Results

### Basic Information

A total of 46 patients with PC-BPPV-cu were enrolled, accounting for 6.36% (46/723) of the BPPV patients during the same period. The ages ranged from 27 to 87 years old, with an average age of 54.91 ± 14.58 years. The peak age of onset was between 50 and 70 years old, accounting for 54.3% (Table 1). The course of the disease was about 12 days. Among them, there were 20 men (43.5%) and 26 women (56.5%). The ratio of men to women was 1:1.3.

**Table 1:** General condition of patients.

**Variables**	**Group A ** **(*n* = 23)**	**Group B** **(*n* = 23)**	***P*-value**
Age, median (IQR)	59 (31)	53 (21)	0.889
Course of disease (day), median (IQR)	12 (8)	9 (11)	0.627
Gender (Male), *n* (%)	8 (34.8)	12 (52.2)	0.234
Atherosclerosis, *n* (%)	16 (69.6)	17 (73.9)	0.743
Smoking, *n* (%)	4 (17.4)	6 (26.1)	0.475
Drinking, *n* (%)	4 (17.4)	8 (34.8)	0.179
Osteoporosis, *n* (%)	12 (52.2)	15 (65.2)	0.369
Mood and sleep disorders, *n* (%)	9 (39.1)	11 (47.8)	0.552
Head trauma, *n* (%)	2 (8.7)	3 (13.0)	1.000
History of abduction vestibule and otology, *n* (%)	13 (56.5)	12 (52.2)	0.767
History of migraine, *n* (%)	4 (17.4)	8 (34.8)	0.179
Immune disease, *n* (%)	7 (30.4)	6 (26.1)	0.743

*IQR, interquartile range*.

Among 46 patients with PC-BPPV-cu, 33 cases (71.7%) were associated with atherosclerosis-related diseases, including 24 cases (52.2%) with hypertension, 22 cases (47.7%) with smoking and/or drinking history, 27 cases with osteoporosis (58.7%), 20 cases (43.5%) with mood and sleep disorder, 5 cases (10.9%) with brain trauma, 25 cases (54.4%) with history of abduction vestibule and otology, 12 cases (26.1%) with migraine, and 13 cases (28.3%) with autoimmune diseases. The abnormality rates on the disease side were as follows: 5 cases with bilateral abnormality, 25 cases with right abnormality, and 16 cases with left abnormality (Table 1). Thereafter, the 46 patients were divided into group A and group B. There was no significant difference in the age, sex ratio, and course of disease between group A and group B.

### Comparison of Characteristics of Nystagmus Induced by PC-BPPV-cu Patients in Different Vertical Positions

**Figure 1:**
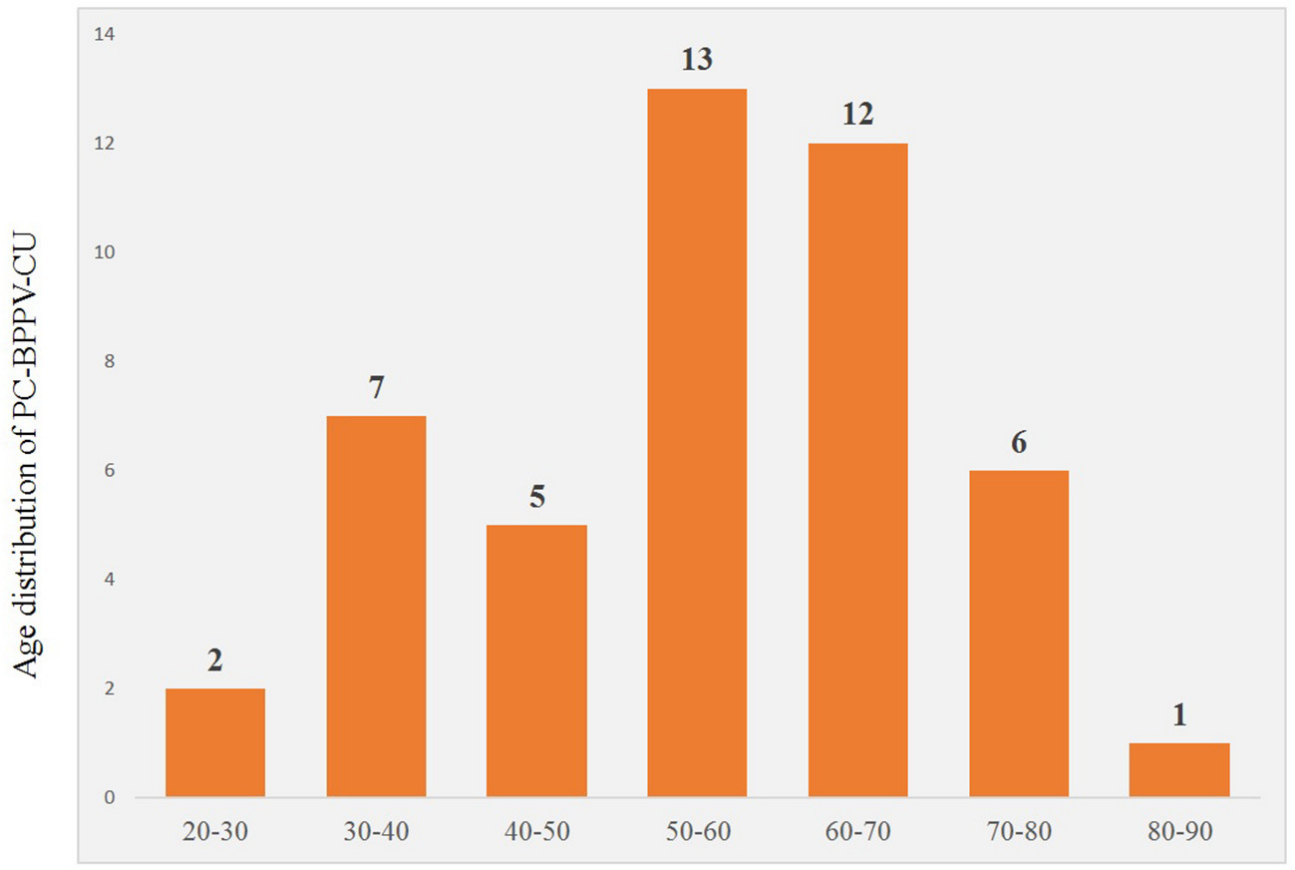
Distribution of torsional nystagmus in three suspension positions in patients with PC-BPPV-cu. UBN, upbeating nystagmus; T-UBN, torsional-upbeating nystagmus.

**Figure 2:**
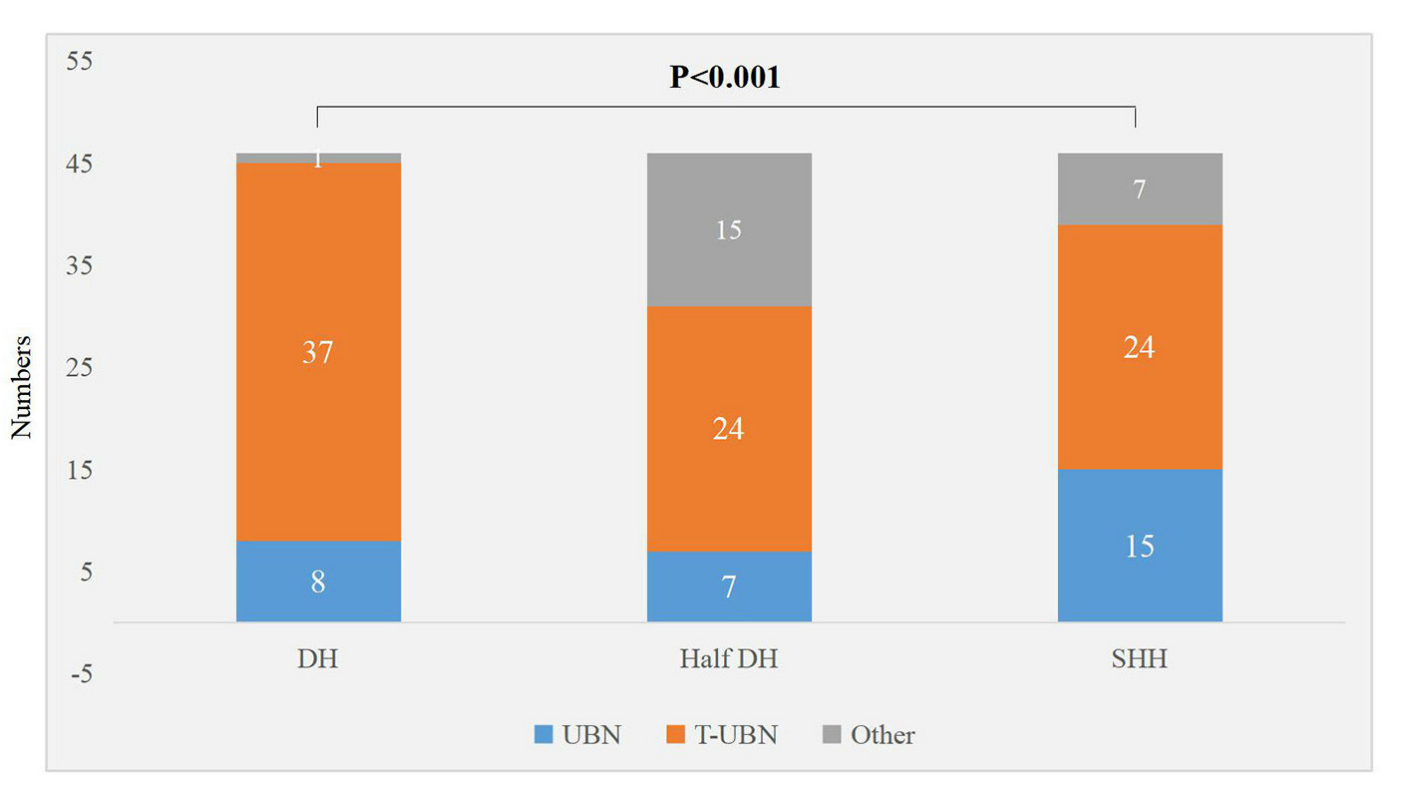
Distribution of sit up and reverse nystagmus in three suspension positions in patients with PC-BPPV-cu.

There were significant differences in the torsional-upbeating nystagmus and upbeating nystagmus among the three supine positions in 46 patients with PC-BPPV-cu (*P* < 0.001) (Table 2, Figure 1). The proportion of torsional-upbeating and upbeating nystagmus in the Dix-Hallpike test is the highest, followed by SHH and least in Half D-HT. The SPV of nystagmus at half D-HT headhanging position was slower than D-HT supine position (*P* < 0.05) and SHH headhanging position (*P* < 0.05). The nystagmus latency of D-HT supine position was significantly shorter than half D-HT suspended head position (*P* < 0.05) and SHH supine position (*P* < 0.05). There was no significant difference in the proportion of PC-BPPV-cu patients with reverse-phase nystagmus direction upon sitting up in three supine positions (*P* > 0.05) (Table 2, Figure 2).

**Table 2:** Results of three types of vertical position induction tests.

**Variables**	**Variable level**	**D-HT**	**Half D-HT**	**SHH**	**Row total**	**Test method**	**Statistics**	***P*-value**
Torsional nystagmus	Upbeating	8 (17.39)	7 (15.22)	15 (32.61)	30 (21.74)	Chi-Squared test	20.646	<0.001
	Upbeating and torsional	37 (80.43)	24 (52.17)	24 (52.17)	85 (61.59)			
	Others	1 (2.17)	15 (32.61)	7 (15.22)	23 (16.67)			
Reverse phase nystagmus	Yes	26 (56.52)	25 (54.35)	23 (50.00)	74 (53.62)	Fisher's exact test	–	0.156
	No	20 (43.48)	16 (34.78)	18 (39.13)	54 (39.13)			
	Without nystagmus	0 (0.00)	5 (10.87)	5 (10.87)	10 (7.25)			
Latency (s)	Mean ± SD	1.53 ± 0.76	2.55 ± 0.83^a^	2.46 ± 0.86^a^	2.13 ± 0.94	One-way Anova analysis	19.075	<0.001
SPV (°/ s)	Median (IQR)	22.0 (17.0, 31.0)	17.0 (10.0, 23.0)^a^	21.0 (15.0, 28.0)^b^	20.0 (15.0, 29.0)	Kruskal-Wallis test	6.923	0.031

*Compared with D-HT, ^a^P <0.05.*

*Compared with Half D-HT, ^b^P <0.05*.

### Evaluation of Vestibular Audiology in Patients With PC-BPPV-cu and Its Correlation With Disease Side

The concordance rates of the four detection methods (canal paresis of caloric test, audiology, cVEMP, vHIT) were similar (χ^2^ = 0.243, *P* = 0.970). Among 46 patients with PC-BPPV-cu, 43 had undergone canal paresis of the caloric test. Among the patients with unilateral semicircular canal paresis, 12 patients (27.9%) were on the right side of semicircular canal paresis, and 7 patients were on the left side (16.3%) (Table 3).

**Table 3:** Cross table of the test results of caloric test, audiology, cVEMP, vHIT, and the condition of the affected side.

**Test methods**	**Results**	**Affected side**, ***n*** **(%)**			**Row total**	**Consistency rate of affected side (%)**
		**Bilateral** **abnormality**	**Right abnormality**	**Left abnormality**		
Caloric test	Bilateral abnormality	2 (50.00)	1 (4.17)	1 (6.67)	4 (9.30)	46.51
	Right abnormality	1 (25.00)	11 (45.83)	0 (0.00)	12 (27.91)	
	Left abnormality	0 (0.00)	0 (0.00)	7 (46.67)	7 (16.28)	
	Normal	1 (25.00)	12 (50.00)	7 (46.67)	20 (46.51)	
Audiology	Bilateral abnormality	3 (60.00)	5 (20.00)	4 (25.00)	12 (26.09)	43.48
	Right abnormality	0 (0.00)	12 (48.00)	1 (6.25)	13 (28.26)	
	Left abnormality	1 (20.00)	1 (4.00)	5 (31.25)	7 (15.22)	
	Normal	1 (20.00)	7 (28.00)	6 (37.50)	14 (30.43)	
cVEMP	Bilateral abnormality	1 (25.00)	4 (26.67)	2 (20.00)	7 (24.14)	41.38
	Right abnormality	1 (25.00)	8 (53.33)	1 (10.00)	10 (34.48)	
	Left abnormality	0 (0.00)	2 (13.33)	3 (30.00)	5 (17.24)	
	Normal	2 (50.00)	1 (6.67)	4 (40.00)	7 (24.14)	
vHIT	Bilateral abnormality	2 (50.00)	4 (44.44)	1 (9.09)	7 (29.17)	41.67
	Right abnormality	1 (25.00)	4 (44.44)	1 (9.09)	6 (25.00)	
	Left abnormality	1 (25.00)	0 (0.00)	4 (36.36)	5 (20.83)	
	Normal	0 (0.00)	1 (11.11)	5 (45.45)	6 (25.00)	

*cVEMP, cervical vestibular evoked myogenic potential; vHIT, video head impulse test*.

All 46 Pc-BPPV-cu patients underwent pure-tone audiometry, of which 14 patients (30.4%) had normal hearing; 20 patients (43.5%) had unilateral hearing loss, and 7 patients (15.2%) had hearing loss on the left side. Among them, there were 13 cases (28.3%) with hearing loss on theright side, and 12 cases (26.1%) with bilateral hearing loss. Among 20 cases with unilateral hearing loss, there were 17 cases (85.0%) which were consistent with the side of the semicircular canal involved in their BPPV, while 3 cases (15.0%) were inconsistent with the side of the involved semicircular canal (Table 3).

During the data collection, the amplitude and latency of the first positive–negative peaks (p13–n23) were calculated from the average of 2 responses to confirm the reproducibility. An asymmetry ratio (AR) for cVEMPs was calculated in order to evaluate the p13-n23 component of the response. We recorded whether cVEMP was abnormal, and the side of the abnormality. The specific evaluation criteria: cVEMP latency wasprolonged, the amplitude was reduced or disappeared, the amplitude AR was >29%, the waveform could not be elicited, and the threshold was raised or lowered as abnormal results (9). Among 46 patients with PC-BPPV-cu, 29 had cVEMP, of which 7 cases (24.1%) had normal cVEMP; 15 cases (51.9%) had unilateral cVEMP abnormality, 7 cases (24.1%) of bilateral cVEMP abnormalities; of 15 cases of unilateral cVEMP abnormalities, 11 cases (73.3%) were consistent with the side of the BPPV involved semicircular canal, and 4 cases (26.7%) were the same as the side of the involved semicircular canal (Table 3).

During the data collection, we recorded whether vHIT was abnormal, and the side of the abnormality. The average gain value of the slow phase of vestibular-ocular reflex (VOR) (the ratio of eye movement velocity and head movement angular velocity at 60 s) can be recorded using vHIT software (10). The specific evaluation criteria were as follows: the normal vestibular eye movement reflex gain range of the horizontal semicircular canal was 0.79–1.20, and the normal range of the vertical semicircular canal was 0.7–1.2. In this study, any one of the following three points was regarded as abnormal vHIT: firstly, the instantaneous vHIT gain value of 60 ms was used as the evaluation index for the horizontal semicircular canal (<0.8 was determined to be abnormal) and the regression gain value was used as the evaluation index for the vertical semicircular canal (<0.7 was determined to be abnormal). Secondly, in 20 vHIT tests, 10 or more compensatory saccade waves (overt saccade or invisible saccade) were judged to be positive. Thirdly, a Bilateral asymmetry ratio >13% was judged to be abnormal. Among 46 patients with PC-BPPV-cu, 24 patients underwent vHIT, of which 6 patients had normal vHIT (25.0%); 11 patients had unilateral vHIT abnormality (45.8%); 7 patients had bilateral vHIT abnormality (29.2%). Among the 11 cases with unilateral vHIT abnormality, 8 cases (72.7%) were consistent with the side of BPPV involved semicircular canal, and 3 cases (27.3%) were inconsistent with the side of the involved semicircular canal (Table 3).

## Discussion

PC-BPPV-cu has received more and more attention in clinical practice. A previous study has found that PC-BPPV-cu accounts for about 6.3% of BPPV patients (11). In this study, we found that PC-BPPV-cu patients accounted for 6.36% (46/723) of the BPPV patients who visited during the same period, which is similar to the results of previous studies and our previous study (6). The male to female ratio of PC-BPPV-cu patients in this study was 1:1.3. There were mostly middle-aged and elderly people. Among patients with unilateral involvement of PC-BPPV-cu, the right-side involvement accounted for 64.3%. The reason is analyzed and the key factors of bone maintenance, bone formation, and bone resorption in middle-aged and elderly women decreasing year by year (12), leading to osteoporosis and abnormal calcium metabolism, affecting endolymph metabolism and inner ear microcirculation (13). Otoliths are deposited on the ampullary crest of the semicircular canal, making it more sensitive to changes in gravity. When the head position was changed, the ampulla cap otoliths of PC-BPPV-cu patients were displaced, resulting in clinical manifestations such as dizziness and nystagmus. Therefore, PC-BPPV-cu is more common in elderly women and is often accompanied by a history of osteoporosis. The high morbidity on the right side is considered to be related to the fact that most patients are used to sleeping on the right side.

The typical nystagmus of PC-BPPV-cu is a nystagmus with a vertical component toward the upper pole of the eyeball and a twist component toward the ground (14). Theoretically, the D-HT test can make the affected crest cap be in a horizontal position, and produce a greater degree of deflection under the action of gravity (8). Our research found that the D-HT test and SHH test had a higher SPV of upbeating nystagmus in patients with PC-BPPV-cu than the Half D-HT test. Compared with the Half D-HT and SHH, D-HT has shorter latency of nystagmus. Carlos et al. (15) pointed out that the latency was an important factor for D-HT in patients with PC-BPPV. It is consistent with our above-mentioned PC-BPPV-cu research. The large-scale changes in spatial position have more obvious physical stimulation to the crest cap otoliths. The crest cap otoliths drive the ampullary crest to deviate to the tube side (producing movement away from the ampullae), resulting in an imbalance of the vestibular tension on both sides. According to Flourens law, the semicircular canal is stimulated to cause the flow of endolymph, and the plane of nystagmus is consistent with the spatial plane of the semicircular canal. In addition, the crest cap is a peripheral sensor that is sensitive to angular acceleration. When the information of the gravity change received by the crest cap reaches a certain threshold and shifts to a certain level, the hair cells are excited to generate action potentials, and further pass through the VOR pathway to cause nystagmus. Cui et al. (16) found that the direction of the torsional component of PC-BPPV is uncertain. If a rotational nystagmus with a vertical upbeating component is induced in the Roll test, it is highly suggested that PC-BPPV may be possible; Hiroaki et al. (17) quantitatively analyzed the horizontal, vertical, and torsional components of nystagmus during the SHH test in patients with PC-BPPV-cu, and found that the SPV of nystagmus torsion in SHH suspension head position was significantly greater than that of nystagmus torsion after sitting up. In our study, although it was impossible to quantitatively compare the torsion component during the vertical position induction test in patients with PC-BPPV-cu, we found that there were significant statistical differences in the proportions of upbeating nystagmus, upbeating and rotational nystagmusin in the three head suspension positions. The proportion of upbeating and rotational nystagmus induced by the D-HT in PC-BPPV-cu patients was higher than that in the Half D-HT, followed by SHH, and least in Half D-HT. Dix Hallpike test is still a classic induction method for PC-BPPV-cu. Half D-HT is simple and easy, especially for patients with neck diseases who cannot look upwards by much. The straight head hanging (SHH) test has obvious significance for the diagnosis of anterior canal benign paroxysmal positional vertigo (18); for patients with PC-BPPV-cu, a roll test can be performed when necessary to help lateral diagnosis. Therefore, Half D-HT and SHH test are powerful supplements to D-HT in the diagnosis of patients with PC-BPPV-cu. The typical nystagmus of PC-BPPV-cu is a nystagmus with a vertical component toward the upper pole of the eye and a torsional component toward the ground (14). The nystagmus of patients with PC-BPPV-cu can usually be induced by vertical position test with vertigo symptoms. The direction of positional nystagmus induced by position test is consistent with the direction of the rotation axis of the stimulated semicircular canal (5). Most of the patients with PC-BPPV-cu in this study were complicated with peripheral vestibular lesions. After imaging examination, the patients in this study have excluded the correlation between positional nystagmus and central vestibular lesions. Patients with central vestibular lesions near the dorsolateral part of the fourth ventricle, dorsal vermis of the cerebellum, nucleus prepositus hypoglossi, cerebellar nodulus, and uvula can show paroxysmal central positional nystagmus in position tests (19, 20). The duration of paroxysmal central positional nystagmus is longer, so the nystagmus of PC-BPPV-cu and paroxysmal central vestibular disorders are often difficult to distinguish. Patients with central vestibular disorders are often accompanied by spontaneous and positional down-beating nystagmus, the direction of positional nystagmus induced by position test was not consistent with the axis of semicircular canal, vertigo may not be associated, and nystagmus cannot be suppressed by fixation. Paroxysmal central positional nystagmus may be ascribed to erroneous neural processing within the velocity-storage circuit that functions in estimating angular head velocity, gravity direction, and inertia (21).

**Figure 3:**
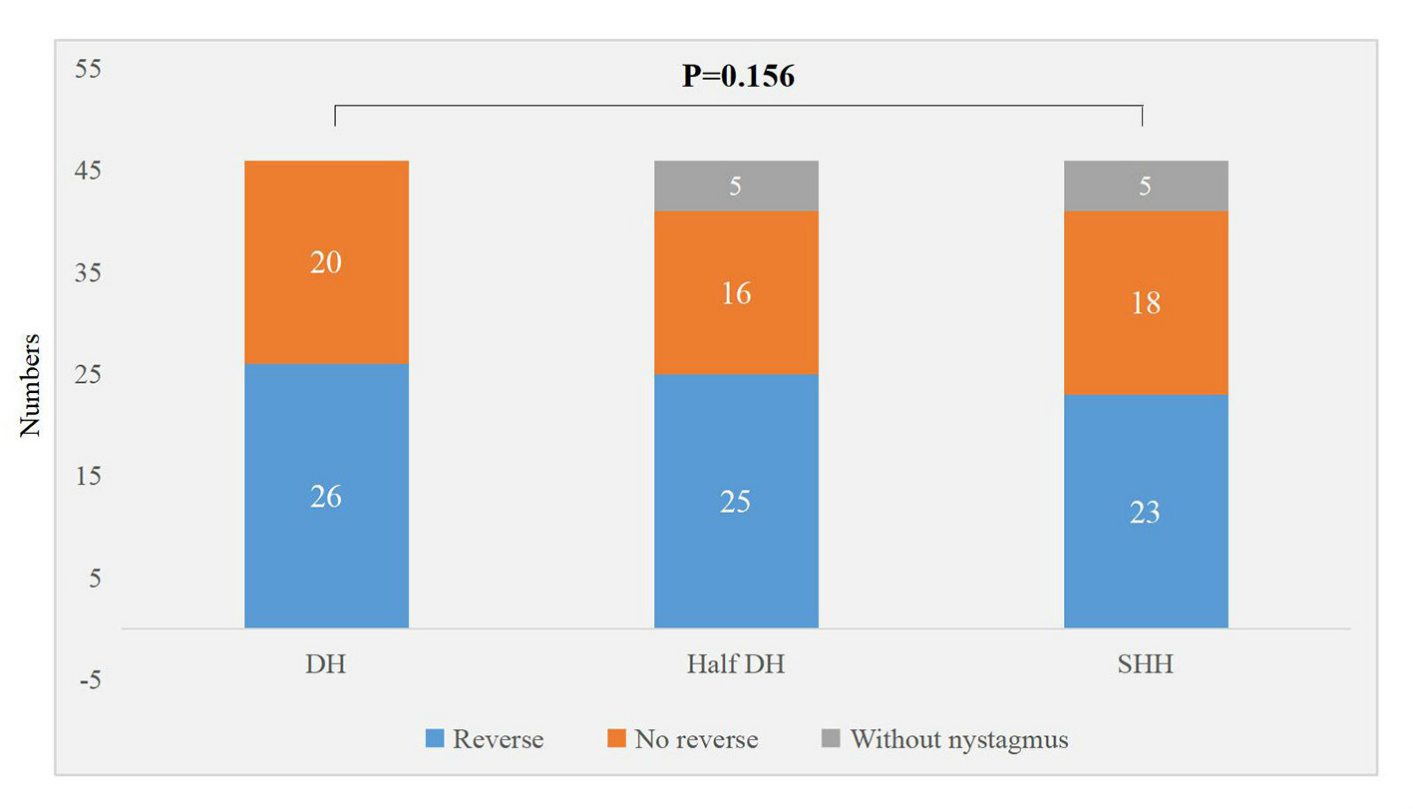
Distribution of sit up and reverse nystagmus in three suspension positions in patients with PC-BPPV-cu.

It is speculated that the generation of reverse phase nystagmus may be related to the semicircular canal inertial rebound of the crest at the abdominal crest (22), there was no significant difference in the proportion of PC-BPPV-cu patients with reverse-phase nystagmus direction upon sitting up in three supine positions (*P* > 0.05) (Table 2, Figure 3), but among the three inducing methods, the proportion of reverse phase nystagmus in D-HT is the highest, D-HT is the most classical and reliable method to diagnose PC-BPPV-cu.

Baloh et al. (17) proposed that the hypofunction of the BPPV semicircular canal was not caused by ectopic otoliths, but by extensive vestibular peripheral organ diseases. Different patients with BPPV have different degrees of damage to the semicircular canal function. The combined application of the cold and heat test and vHIT test could objectively and comprehensively evaluate the semicircular canal function of patients with idiopathic BPPV. Bi et al. (23) found that the semicircular canal paresis rate of BPPV patients was 57%. Nakahara et al. (24) found that the abnormal rate of oVEMP in BPPV patients was higher than that of the normal population. We previously reported that unilateral vestibular dysfunction and audiological abnormalities assisted the positioning of multitubular BPPV (7). In this present study, most patients with PC-BPPV-cu had semicircular canal paresis and abnormalities in audiology, cVEMP, and vHIT. The hearing test is used as a routine examination and observation index for BPPV patients. There are many influencing factors, such as age, disease histories, and the patient's own subjective perception of hearing, etc. Therefore, for patients with PC-BPPV-cu, a combined application of semicircular canals and otolith function evaluation is helpful for the diagnosis and etiological treatment of patients with PC-BPPV-cu.

The sample size of this study is relatively small. Future work should expand the sample size and analyze the clinical characteristics of PC-BPPV-cu patients, such as a comparison of the correlation between the subtypes of different sleep disorders and the incidence of PC-BPPV-cu.

In summary, the Half D-HT is simple and feasible but might bring a risk of false-negatives to diagnosis of torsional-upbeating nystagmus and upbeating nystagmus. The D-HT is still a classic induction method for PC-BPPV-cu. The two complement each other and may aid in the diagnosis of PC-BPPV-cu patients. Future clinical applications of Half D-HT require extensive research to determine its diagnostic efficacy.

In addition, for patients with PC-BPPV-cu, we can combine audiology, semicircular canal and otolitic device function evaluation technology to assist patients' diagnoses. The diagnosis of PC-BPPV-cu could guide the medication and rehabilitation of patients with PC-BPPV-cu, and correctly assess the prognosis.

## Data Availability Statement

The original contributions presented in the study are included in the article/supplementary material, further inquiries can be directed to the corresponding author/s.

## Ethics Statement

The studies involving human participants were reviewed and approved by the First Hospital of Hebei Medical University. The patients/participants provided their written informed consent to participate in this study.

## Author Contributions

WW, PG, and SY have made substantial contributions to the conception, design of the study, and have been involved in drafting the manuscript. S and RH were involved in the acquisition of data, data entry, and data cleaning. DL, YLiu, and TZ were involved in the analysis and interpretation of data. SL, YW, and YLi were involved in revising the manuscript critically for important intellectual content. YW made contributions to data collection and analysis. All authors contributed substantially to its revision. All authors read and approved the final manuscript.

## Funding

This study was funded by Hebei Provincial Health Commission (No. 20220174).

## Conflict of Interest

The authors declare that the research was conducted in the absence of any commercial or financial relationships that could be construed as a potential conflict of interest.

## Publisher's Note

All claims expressed in this article are solely those of the authors and do not necessarily represent those of their affiliated organizations, or those of the publisher, the editors and the reviewers. Any product that may be evaluated in this article, or claim that may be made by its manufacturer, is not guaranteed or endorsed by the publisher.
